# Supplementary material for: Assessment Tools to Examine Illness Understanding in Patients with Advanced Cancer: A Systematic Review of Randomized Clinical Trials
Source: Cancers (Basel). 2025 Jan 24;17(3):385. doi: 10.3390/cancers17030385 (PMC11816152; doi:10.3390/cancers17030385)
Supplement: Supplementary file 1 [file cancers-17-00385-s001.zip › cancers-3379995-supplementary.pdf]

Assessment Tools to Examine Illness Understanding in Patients with Advanced Cancer: A Systematic Review of Randomized Clinical Trials

Section S1: Ovid Medline, Ovid Embase, and Web of Science Search Strings

Ovid Medline and Ovid Embase Search String

1. Patient/ or Patients/
2. Exp Cancer Patient/
3. Acutely Ill Patient/
4. Critically Ill Patient/
5. Exp Hospital Patient/
6. Terminally Ill Patient/ or Terminally Ill/
7. (Patient*).ti,ab.
8. OR/1-7
9. Neoplasm Metastasis/ or Advanced Cancer/
10. ((advanced or incurable or "late stage" or metastatic or metastas* or untreatable or recurrent or secondary or terminal or uncurable or "stage 4" or "stage IV") ADJ2 (cancer* or tumor* or tumour* or "bone cancer" or "breast cancer" or "colon cancer" or "colorectal cancer" or "kidney cancer" or "liver cancer" or "lung cancer" or malignan* or "non-small cell lung cancer" or nsclc or "pancreatic cancer" or "prostate cancer")).ti,ab.
11. (advanced ADJ (adenocarcinoma* or blastoma* or cancer* or carcinoma* or chondrosarcoma* or choriocarcinoma* or glioma* or hepoblastoma* or leukemia or leukaemia or leiomyosarcoma* or liposarcoma* or lymphoma* or malignan* or medulloblastoma* or melanoma* or mesothelioma* or myeloma* or myosarcoma* or myxosarcoma* or neoplas* or neuroblastoma* or NSCLC or osteosarcoma* or papillomatosis or paraganglioma* or pheochromocytoma or retinoblastoma* or rhabdomyosarcoma* or sarcoma* or SCLC)).ti,ab.
12. OR/9-11
13. Prognosis/ or Cancer Prognosis/
14. Cancer Diagnosis/ or Diagnosis/
15. Disease Course/
16. Disease Severity/ or Patient Acuity/
17. Terminal Disease/
18. Exp Treatment Outcome/
19. Life Expectancy/
20. Decision Making/ or Patient Decision Making/
21. curability.ti,ab.
22. (goal* ADJ1 (care or discussion or therapy or treatment or treatment or therapy)).ti,ab.
23. Illness.ti,ab.
24. "life expectancy".ti,ab.
25. (prognosis or prognostic).ti,ab.
26. (risks ADJ1 benefits).ti,ab.
27. (status or treatment).ti,ab.
28. OR/13-27
29. Comprehension/
30. Expectation/
31. Perception/
32. Patient Education/ or "Patient Education as Topic"/

33. "Health Knowledge, Attitudes, Practice"/
34. (understand* or understood or comprehension or comprehend*).ti,ab.
35. knowledge.ti,ab.
36. awareness.ti,ab.
37. (expectation*).ti,ab.
38. (perception* or realism or realistic or uncertain* or unrealistic).ti,ab.
39. conception.ti,ab.
40. discussion*.ti,ab.
41. OR/29-40
42. exp "Surveys and Questionnaires"/ or Exp Questionnaire/ or Open Ended Questionnaire/ or Structured Questionnaire/
43. Interview/ or "Semi Structured Interview"/ or exp Telephone Interview/ or Unstructured Interview/ or Video Interview/ or "Interview as Topic"/
44. Assessment/ or Multiple Choice Test/
45. "Datasets as Topic"/ or Data Collection Method/
46. Validity/ or Validation Study/ or "Validation Studies as Topic"/
47. ((screen* or evaluat* or assess* or diagnos* or rating or rate or measur*) adj2 (index or test* or instrument* or inventor* or battery or batteries or tool* or scale* or checklist* or check list* or schedule*)).ti,ab.
48. (analysis or assess* or batter* or checklist* or "coding manual*" or "coding scheme*" or discussion* or evaluat* or framework or index or indicator* or instrument* or interview* or inventor* or measure or measures or measurement or measuring or measuring or posttest* or "post-test*" or pretest* or "pre-test*" or rate or rating or questionnaire* or rating or scale or scales or schedule* or score or scores or scoring or scenario*or screen or screening or standard* or subscale* or survey* or test or tested or tests or tool or tools or vignette *).ti,ab.
49. OR/42-48
50. AND/8,12,28,41,49
51. randomized controlled trial/
52. controlled clinical trial/
53. (randomised or randomized).ti,ab.
54. placebo.ab.
55. clinical trials as topic.sh.
56. randomly.ab.
57. trial.ti.
58. Randomized Controlled Trials as Topic/ or " randomized controlled trial (topic)"/
59. ("phase 3*" or "phase III*").ti,ab.
60. (ANZCTR or ChiCTR or clinicaltrials or CRiS or CTRI or DRKS or EUDRACT or IRCT or ISRCTN or JPRN or NTR or PACTR or ReBec or RPCEC or SLCTR or TCTR or UMIN or UMIN CTR or URCC).si. and (randomized or randomised).ti,ab.
61. OR/51-60
62. AND/50,61
63. Abstract Report/ or Academic Dissertation/ or "Academic Dissertation As Topic"/ or Book/ or Case Finding/ or Case Report/ or Case Study/ or Exp Clinical Protocol/ or Clinical Trial Protocol/ or Comment/ or Conference Abstract/ or Conference Paper/ or Congress/ or Exp "Congresses As Topic"/ or Editorial/ or English Abstract/ or Exp Historical Article/ or Exp "In Vitro Study"/ or Letter/ or Exp Meta Analysis/ or "Meta Analysis (Topic)"/ or Note/ or Overall/ or Pilot Study/ or Exp Practice Guideline/ or Review/ or Symposium/ or Systematic Review/ or "Systematic Review (Topic)"/ or Validation Study/ or "Video-Audio Media"/ or Workshop/

64. (abstract* or "annual meeting" or "case report" or comment* or conference or congress or dissertation* or editor* or feasibility or letter or papers or poster or proceeding* or protocol or symposium or validation or systematic review or systematic literature review or meta-analysis).ti,ab
65. OR/63-64
66. 62 NOT 65
67. Limit 66 to English
68. Limit 67 to human
69. (amphibian* or animal? or bird or birds or bovine or canine or cat or cats or cattle or cow or cows or dog or dogs or ferret* or fish or frog* or "guinea pig*" or hamster* or horse* or "in vitro" or lambs or llama* or marmoset* or mice or monkey* or mouse or murine or pig or pigs or piglets or porcine or primate or primates or rabbit* or rat or rats or sheep or swine or trout* or veterinar* or zebra*).ti.
70. 68 NOT 69

Web of Science Search String

TOPIC: patient* NEAR/3 (decision* or decid*)

TOPIC: (advanced or incurable or "late stage" or metastatic or metastas* or untreatable or recurrent or secondary or terminal or uncurable or "stage 4" or "stage IV") NEAR/2 (adenocarcinoma* or blastoma* or "bone cancer" or "breast cancer" or cancer* or carcinoma* or chondrosarcoma* or choriocarcinoma* or "colon cancer" or "colorectal cancer" or glioma* or hepoblastoma* or "kidney cancer" or leukemia or leukaemia or leiomyosarcoma* or liposarcoma* or "liver cancer" or "lung cancer" or lymphoma* or malignan* or medulloblastoma* or melanoma* or mesothelioma* or myeloma* or myosarcoma* or myxosarcoma* or neoplas* or neuroblastoma* or "non-small cell lung" or NSCLC or osteosarcoma* or "pancreatic cancer" or papillomatosis or paraganglioma* or pheochromocytoma or "prostate cancer" or retinoblastoma* or rhabdomyosarcoma* or sarcoma* or SCLC or "thyroid cancer")

TOPIC: awareness or comprehension or comprehend* or conception or discussion* or expectation* or knowledge or perception* or realism or realistic or uncertain* or unrealistic or understand* or understood or (goal* NEAR/1 (care or discussion or therapy or treatment or treatment or therapy or Illness or curability or "life expectancy" or prognosis or prognostic or status or treatment) or (risks NEAR/2 benefits))

TOPIC: (random* or controlled or clinical) NEAR/2 trial* awareness or comprehension or comprehend* or conception or discussion* or expectation* or knowledge or perception* or realism or realistic or uncertain* or unrealistic or understand* or understood or (goal* NEAR/1 (care or discussion or therapy or treatment or treatment or therapy or Illness or curability or "life expectancy" or prognosis or prognostic or status or treatment) or (risks NEAR/2 benefits))

41. OR/29-40
42. exp "Surveys and Questionnaires"/ or Exp Questionnaire/ or Open Ended Questionnaire/ or Structured Questionnaire/
43. Interview/ or "Semi Structured Interview"/ or exp Telephone Interview/ or Unstructured Interview/ or Video Interview/ or "Interview as Topic"/
44. Assessment/ or Multiple Choice Test/
45. "Datasets as Topic"/ or Data Collection Method/

46. Validity/ or Validation Study/ or "Validation Studies as Topic"/
47. ((screen* or evaluat* or assess* or diagnos* or rating or rate or measur*) adj2 (index or test* or instrument* or inventor* or battery or batteries or tool* or scale* or checklist* or check list* or schedule*)).ti,ab.
48. (analysis or assess* or batter* or checklist* or "coding manual*" or "coding scheme*" or discussion* or evaluat* or framework or index or indicator* or instrument* or interview* or inventor* or measure or measures or measurement or measuring or measuring or posttest* or "post-test*" or pretest* or "pre-test*" or rate or rating or questionnaire* or rating or scale or scales or schedule* or score or scores or scoring or scenario*or screen or screening or standard* or subscale* or survey* or test or tested or tests or tool or tools or vignette *).ti,ab.
49. OR/42-48
50. AND/8,12,28,41,49
51. randomized controlled trial/
52. controlled clinical trial/
53. (randomised or randomized).ti,ab.
54. placebo.ab.
55. clinical trials as topic.sh.
56. randomly.ab.
57. trial.ti.
58. Randomized Controlled Trials as Topic/ or " randomized controlled trial (topic)"/
59. ("phase 3*" or "phase III*").ti,ab.
60. (ANZCTR or ChiCTR or clinicaltrials or CRiS or CTRI or DRKS or EUDRACT or IRCT or ISRCTN or JPRN or NTR or PACTR or ReBec or RPCEC or SLCTR or TCTR or UMIN or UMIN CTR or URCC).si. and (randomized or randomised).ti,ab.
61. OR/51-60
62. AND/50,61
63. Abstract Report/ or Academic Dissertation/ or "Academic Dissertation As Topic"/ or Book/ or Case Finding/ or Case Report/ or Case Study/ or Exp Clinical Protocol/ or Clinical Trial Protocol/ or Comment/ or Conference Abstract/ or Conference Paper/ or Congress/ or Exp "Congresses As Topic"/ or Editorial/ or English Abstract/ or Exp Historical Article/ or Exp "In Vitro Study"/ or Letter/ or Exp Meta Analysis/ or "Meta Analysis (Topic)"/ or Note/ or Overall/ or Pilot Study/ or Exp Practice Guideline/ or Review/ or Symposium/ or Systematic Review/ or "Systematic Review (Topic)"/ or Validation Study/ or "Video-Audio Media"/ or Workshop/
64. (abstract* or "annual meeting" or "case report" or comment* or conference or congress or dissertation* or editor* or feasibility or letter or papers or poster or proceeding* or protocol or symposium or validation or systematic review or systematic literature review or meta-analysis).ti,ab
65. OR/63-64
66. 62 NOT 65
67. Limit 66 to English
68. Limit 67 to human
69. (amphibian* or animal? or bird or birds or bovine or canine or cat or cats or cattle or cow or cows or dog or dogs or ferret* or fish or frog* or "guinea pig*" or hamster* or horse* or "in vitro" or lambs or llama* or marmoset* or mice or monkey* or mouse or murine or pig or pigs or piglets or porcine or primate or primates or rabbit* or rat or rats or sheep or swine or trout* or veterinar* or zebra*).ti.
70. 68 NOT 69
